# Enhancing Emergency Trauma Management Skills Through Simulation: A Quality Improvement Initiative in Ghana

**DOI:** 10.7759/cureus.105156

**Published:** 2026-03-13

**Authors:** Nkechi O Dike, Priscilla Okyere, Jonathan Kajjimu, Emmanuel K Achampong

**Affiliations:** 1 Maersk Clinical Skills Laboratory, University of Cape Coast School of Medical Sciences, Cape Coast, GHA; 2 Faculty of Medicine, Mbarara University of Science and Technology, Mbarara, UGA; 3 Medical Education and Information Technology, University of Cape Coast School of Medical Sciences, Cape Coast, GHA

**Keywords:** continuing professional development, emergency care, ghana, quality improvement, simulation-based training, trauma, workforce capacity

## Abstract

Introduction

In Ghana, persistent gaps in emergency and trauma care are linked to limited training opportunities and uneven workforce distribution. This pilot program evaluated the feasibility and outcomes of a regional, simulation-based quality improvement initiative for emergency trauma care in the Central Region of Ghana.

Methods

A pre- and post-test pilot was conducted as part of a regional quality improvement effort among multidisciplinary clinicians of various facilities. Two simulation-based continuous professional development (CPD) modules, Basic Emergency and Trauma Management and Suture Techniques & Wound Care, were delivered. Quantitative data were analyzed using chi-square tests (p<0.05), and open-ended responses were analyzed thematically.

Results

The majority of participants were nurses (51.1%, n=24) and from district hospitals (61.7%, n=29). Additionally, 53.2% (n=25) of facilities reported functional resuscitation areas, and 27.7% (n=13) had access to blood transfusion services. Knowledge scores improved significantly across all domains, from a mean of 52.6% pre-training to 85.9% post-training (p<0.001). The largest gains were in spinal precautions, focused abdominal sonography in trauma (FAST) interpretation, and bite wound management. All participants expressed high satisfaction, citing the realism and interactivity of the simulation as major strengths, and recommended integrating simulation into regular CPD. Key challenges reported included limited trauma equipment (76.6%, n=36), staffing shortages (66.0%, n=31), and weak referral systems (51.1%, n=24).

Conclusion

This pilot demonstrated that simulation-based training is both feasible and effective for building emergency and trauma care competence in resource-limited settings. Embedding simulation-based CPD within regional quality improvement frameworks offers a scalable strategy for workforce development and health system strengthening in Ghana.

## Introduction

Quality of healthcare is central to achieving universal health coverage (UHC) and sustainable development goal three (SDG 3) [1,2]. High-quality care must be safe, effective, timely, efficient, integrated, people-centered, and equitable [3,4]. Achieving this requires more than physical access; it depends on a competent health workforce capable of delivering evidence-based care across all service levels [5]. Workforce competence is therefore a cornerstone of quality improvement (QI), as provider performance directly influences patient safety and outcomes [6]. In many low- and middle-income countries (LMICs), including Ghana, gaps in emergency and trauma care quality stem from limited training opportunities, mal-distribution of skilled health workers, and variable exposure to acute cases [7,8]. These inequities are further exacerbated by skill decay over time, especially among clinicians in peripheral or rural facilities where acute cases are infrequent and opportunities for refresher training are scarce. Such disparities in competence contribute to delays in diagnosis, suboptimal stabilization, and avoidable mortality [9,10].

Simulation-based training (SBT) has emerged globally as an effective and adaptable approach to strengthening competence and teamwork across clinical settings [11,12]. It provides safe, hands-on environments where learners can practice critical procedures, receive immediate feedback, and build both technical and non-technical skills without risk to patients [13]. Systematic reviews and regional experiences in sub-Saharan Africa demonstrate that simulation, when contextualized and supported, improves knowledge, technical performance, and communication and can even inform QI processes by revealing system-level bottlenecks [14,15].

However, simulation remains underutilized in Ghana's healthcare system due to resource limitations, lack of trained facilitators, and the absence of structured, accredited programs that link education directly with system performance improvement [16,17]. However, when implemented as part of a QI process, simulation can serve a dual function of strengthening individual clinical competence and generating insights into broader system needs [18,19].

Against this background, we piloted a regional, simulation-based QI initiative in the central region of Ghana. The program sought to translate regional morbidity and mortality priorities into targeted continuous professional development (CPD) modules that could be delivered in a hands-on, contextually relevant manner and scaled if feasible. The pilot comprised two modules, namely, Basic Emergency Trauma and Fracture Management and Suture Techniques & Wound Care, both delivered as accredited CPD activities at the Maersk Clinical Skills and Simulation Laboratory, University of Cape Coast (UCC), in partnership with the Central Regional Health Directorate. This paper describes the implementation, evaluation, and outcomes of this initiative, focusing on short-term knowledge and confidence gains, feasibility, and implications for future scale-up and health system strengthening.

## Materials and methods

Study design and setting

This was a pragmatic, pre-post quasi-experimental QI pilot conducted in the Central Region of Ghana. It was implemented collaboratively by the UCC School of Medical Sciences and the Central Regional Health Directorate, as part of ongoing regional efforts to enhance emergency and trauma care delivery and overall improved health outcomes in the region. Training took place at the Maersk Clinical Skills and Simulation Laboratory, UCC, using a combination of high-fidelity manikins (SimMan 3G Plus; Laerdal Medical, Stavanger, Norway), task trainers, and low-fidelity models appropriate for basic procedures.

The pilot formed part of a broader QI strategy designed to strengthen emergency and trauma care capacity at the district and sub-district levels, where timely and competent stabilization is often the determinant of survival.

Participants

Fifty multidisciplinary healthcare workers were nominated by the Regional Health Directorate to represent facilities across the region, including district hospitals, health centers, and community-based health planning and services (CHPS) compounds. Participants included doctors, nurses, and physician assistants involved in frontline patient care. Participant selection was purposive to ensure diversity of cadres and equitable representation of facility types.

Training intervention

Two modules were developed and delivered using experiential learning and simulation-based education principles: (1) Basic Emergency Trauma and Fracture Management and (2) Suture Techniques & Wound Care. Together, acronymed as the BET Program, the modules were accredited for CPD credits by the Medical and Dental Council of Ghana. Each module combined short, focused didactic sessions to standardize decision-making and clinical reasoning; hands-on skills practice using task trainers and manikins for procedures such as airway management, fracture immobilization, and suturing; and team-based simulation scenarios that integrated case recognition, triage, and stabilization. Structured debriefings followed each simulation, allowing participants to reflect on clinical reasoning, communication, and teamwork under pressure. Faculty comprised emergency physicians, surgical educators, nurse trainers, and simulation specialists from UCC and partnering facilities. The two-day format was intentionally designed to minimize service disruption while maximizing hands-on learning and feedback.

Data collection

Participants completed pre- and post-training surveys using Google Forms (Google, Mountain View, CA) accessed via QR codes. The pre- and post-training survey questionnaires were developed by the training team specifically for this training initiative, to align with the specific skills addressed in the Basic Emergency Trauma (BET) course. The content was informed by regional morbidity and mortality priorities and by the practical competencies targeted during the hands-on sessions, supported with literature reviews. The questionnaires were reviewed by faculty members for clarity and relevance before implementation. The surveys assessed demographic data, baseline knowledge, and self-rated confidence across defined topic areas using multiple-choice and Likert-scale questions. Additional items evaluated participants' facility resources (availability of theatre, laboratory, blood bank, ambulance, and imaging services) and included open-ended questions to capture qualitative reflections, such as current challenges in trauma care and priority areas for future training. Participation was voluntary and anonymous, with consent through survey completion.

Data analysis

Data were exported from Google Forms into Microsoft Excel (2016 MSO, version 16.0.4456.1003; Microsoft® Corp., Redmond, WA) for cleaning and statistical analysis. Quantitative data were analyzed descriptively with frequencies and percentages. Chi-square tests (χ²) were used to compare pre- and post-training responses, with statistical significance set at p < 0.05. Qualitative data from open-ended questions were analyzed thematically to identify common challenges, learning experiences, and perceived system gaps.

Ethical considerations

This initiative was conducted as a QI activity under the joint oversight of the Central Regional Health Directorate and the UCC School of Medical Sciences. The Department of Medical Education & Information Technology approved the study as not involving human-subjects research. Hence, formal institutional review board approval was not required. All participants gave informed consent for inclusion in the training and feedback components, and no personal identifiers were collected.

## Results

Participant characteristics

Of the 50 clinicians who participated, 47 (94%) completed both assessments. Participants represented diverse cadres and practice settings across the central region. Most respondents were nurses (24, 51.1%), followed by medical officers (13, 27.7%), physician assistants (7, 14.9%), and others, including midwives and emergency medical technicians (EMTs) (3, 6.3%). Most participants worked in district hospitals (29, 61.7%), followed by health centers (10, 21.3%), regional or teaching hospitals (5, 10.6%), and private facilities (3, 6.4%). Participants had a median of five years of clinical experience, and 70% reported encountering trauma or emergency cases at least weekly (Table 1).

**Table 1:** Demographic and Professional Characteristics of Participants (n=47) EMTs: emergency medical technicians

Characteristic	Category	n (%)
Profession/Cadre	Nurse	24 (51.1)
	Medical Officer	13 (27.7)
	Physician Assistant	7 (14.9)
	Others (Midwives, EMTs)	3 (6.3)
Type of Facility	District Hospital	29 (61.7)
	Health Center	10 (21.3)
	Regional/Teaching Hospital	5 (10.6)
	Private Facility	3 (6.4)
Years of Clinical Experience	1-3 years	11 (23.4)
	4-6 years	17 (36.2)
	7-10 years	10 (21.3)
	>10 years	9 (19.1)
Frequency of Encountering Trauma Cases	Daily	8 (17.0)
	Weekly	25 (53.2)
	Monthly	10 (21.3)
	Rarely	4 (8.5)

Facility resources

Participants were asked to indicate which essential trauma-care resources were available at their facilities. Only 25 (53.2%) participants reported having access to a functional resuscitation area or emergency room (ER), and 28 (59.6%) had a functional operating theatre. Laboratory services were the most consistently available (30, 63.8%). Blood bank services (13, 27.7%) and reliable oxygen supply (21, 44.7%) were limited. Imaging capacity was low: X-ray (17, 36.2%), ultrasound (15, 31.9%), and computed tomography (CT) (1, 2.1%). Only 18 (38.3%) reported ambulance availability, and 20 (42.6%) had essential trauma equipment (Table 2).

**Table 2:** Availability of Trauma and Emergency Care Resources at Participants' Facilities

Facility Resource	Available, n (%)	Not Available, n (%)
Functional Resuscitation Area/Emergency Room	25 (53.2)	22 (46.8)
Theatre/Operating Room	28 (59.6)	19 (40.4)
Blood Bank/Transfusion Services	13 (27.7)	34 (72.3)
Laboratory with Emergency Testing	30 (63.8)	17 (36.2)
X-ray Machine	17 (36.2)	30 (63.8)
Ultrasound Machine	15 (31.9)	32 (68.1)
CT Scan	1 (2.1)	46 (97.9)
Reliable Oxygen Supply	21 (44.7)	26 (55.3)
Ambulance or Transport System	18 (38.3)	29 (61.7)
Essential Trauma Equipment (Splints, Collars, Suction, etc.)	20 (42.6)	27 (57.4)

Knowledge and skill outcomes

Participants demonstrated marked improvement across all assessed domains following training. Correct responses increased from an average of 52.6% (pre-training, n=47) to 85.9% (post-training, n=47), representing a 33.3 percentage-point improvement (Table 3).

**Table 3:** Pre- and Post-Training Knowledge and Skills Assessment Δ: percentage difference between pre- and post-training assessments; df: degree of freedom; FAST: focused assessment with sonography in trauma

Topic Area	Pre-training, n (%)	Post-training, n (%)	Δ (%)	χ² (df=1)	p-value
Airway Management	27 (57.4)	43 (91.5)	34.1	10.9	0.001
Tension Pneumothorax Recognition	24 (51.1)	41 (87.2)	36.1	11.7	<0.001
Control of External Bleeding	29 (61.7)	42 (89.4)	27.7	8.4	0.004
Pelvic Stabilization	23 (48.9)	37 (78.7)	29.8	8	0.005
Fracture Immobilization	26 (55.3)	40 (85.1)	29.8	8.6	0.003
Spinal Precautions	21 (44.7)	39 (83.0)	38.3	10.5	0.001
FAST/Ultrasound Interpretation	15 (31.9)	33 (70.2)	38.3	9.8	0.002
Referral and Communication	32 (68.1)	43 (91.5)	23.4	6.6	0.01
Wound Cleansing & Debridement	25 (53.2)	41 (87.2)	34	10.8	0.001
Bite Wound Management	22 (46.8)	39 (83.0)	36.2	10.9	0.001
Overall Mean	-52.6	-85.9	33.3	-	<0.001

Perceptions of training and confidence

Participants rated the training very highly; 46 (97.9%) found the hands-on sessions extremely helpful, 44 (93.6%) reported improved understanding of stabilization and referrals, and all respondents (100%) were satisfied with facilitators and recommended integrating simulation regularly into CPDs.

Challenges in managing trauma cases

Participants identified multiple barriers, including limited trauma/resuscitation equipment (36, 76.6%), inadequate staffing (31, 66.0%), lack of blood transfusion services (28, 59.6%), poor referral systems (24, 51.1%), limited access to diagnostics (21, 44.7%), infrequent trauma-specific training (20, 42.6%), and inconsistent availability of essential emergency drugs (14, 29.8%) (Table 4).

**Table 4:** Reported Challenges in Managing Trauma Cases

Challenge Area	n (%)
Limited Trauma/Resuscitation Equipment	36 (76.6)
Inadequate Staffing/Manpower	31 (66.0)
Lack of Blood or Transfusion Services	28 (59.6)
Delayed or Unsafe Referral Systems	24 (51.1)
Limited Access to Imaging/Diagnostics	21 (44.7)
Infrequent Trauma-Specific Training	20 (42.6)
Poor Inter-facility Communication	17 (36.2)
Inconsistent Availability of Emergency Drugs	14 (29.8)

Open-ended reflections and future needs

Participants described the course as practical, engaging, and confidence-building. Many noted that the simulation exposed hidden practice gaps. Requested future modules included obstetric and pediatric emergencies, advanced trauma care, burns, and mass casualty management.

## Discussion

This pilot demonstrated that a short, simulation-based CPD intervention can produce substantial and immediate gains in emergency and trauma care competence among frontline clinicians in a resource-limited region of Ghana. The improvement in knowledge from 52.6% at baseline to 85.9% post-training aligns with evidence that simulation-based education produces superior learning outcomes compared with traditional didactic teaching, particularly for high-risk, time-critical clinical skills [20-22]. Studies from other LMICs similarly show that simulation improves emergency care performance, procedural competence, and clinical decision-making [23-25]. These results reaffirm simulation as a key strategy for strengthening emergency care capacity where patient volumes, supervision, or case mix are insufficient for consistent experiential learning.

The strongest gains in this study occurred in domains such as spinal precautions, focused abdominal sonography in trauma (FAST) interpretation, and bite-wound management, skills essential for early trauma evaluation. Similar improvements have been reported in LMIC simulation programs focused on trauma resuscitation, obstetric emergencies, and pediatric acute care [24,25]. High participant satisfaction mirrors findings that simulation engages learners more deeply than lectures and enhances retention and confidence [21]. The multi-professional approach also reflects best practices: team-based simulation improves communication, role clarity, and adherence to critical actions during resuscitation [26].

While the intervention improved competence, it also illuminated persistent system-level constraints. Equipment shortages (reported by 76.6% of participants), limited staffing (66.0%), and inconsistent referral pathways mirror findings of national and regional assessments of emergency care readiness in Ghana and comparable LMIC settings [7,10,11,27]. Fewer than 30% of facilities in this study reported access to a blood bank, consistent with prior research documenting major gaps in the trauma care chain [7]. Simulation served as an important diagnostic tool, revealing operational bottlenecks such as delayed escalation, fragmented communication, and inconsistent triage practices. This aligns with literature showing that simulation exposes latent safety threats and system vulnerabilities not easily captured through routine audits [28].

The findings in this study highlight the relevance of simulation for CPD and continuous quality improvement. Competency-based training models, especially those grounded in experiential learning, are widely recommended for emergency care development in LMICs [29,30]. For generalist clinicians who often shoulder emergency responsibilities with limited support, standardized simulation-based CPD can reduce variation in practice and reinforce critical actions across facilities. Structured debriefings, a core feature of this intervention, are recognized as powerful tools for reflective learning and performance improvement.

Simulation also supports broader system strengthening. Evidence shows that simulation-integrated QI cycles can improve adherence to clinical protocols, enhance teamwork, and strengthen safety culture [28]. Embedding simulation within district and regional health strategies may therefore improve both clinical competence and operational readiness.

This pilot demonstrates a feasible and replicable model for scaling simulation-based CPD in Ghana. The program leveraged existing infrastructure, required modest equipment, and successfully trained a multidisciplinary cohort. These are elements that support scalability. Similar national-scale implementations have been achieved in Rwanda, Tanzania, and Ethiopia through integration of simulation into CPD requirements, mentorship structures, and regional QI initiatives [23,30]. Aligning this model with Ghana's CPD policies and regional emergency care strengthening efforts could broaden its impact.

## Conclusions

This pilot demonstrated that a regionally delivered, simulation-based CPD program can meaningfully strengthen emergency and trauma care competence among frontline clinicians in Ghana. Integrating simulation-based CPD into regional quality improvement strategies offers a feasible and scalable pathway for strengthening Ghana's emergency care workforce. As national CPD policies continue to evolve, embedding structured simulation within routine professional development, mentorship, and facility-level QI cycles could help standardize clinical practice, enhance teamwork, and improve patient safety.

Importantly, our initiative also highlighted an additional utility of simulation as a diagnostic tool for health system improvement. Addressing identified health system gaps will be essential to achieving sustained improvements in emergency and trauma care. Scaling this model while simultaneously addressing the resource and referral deficiencies identified has strong potential to contribute to a more resilient, prepared, and equitable emergency care system in Ghana.
